# Comparative safety and efficacy of topical mometasone furoate with other topical corticosteroids

**DOI:** 10.1111/ajd.12762

**Published:** 2018-02-07

**Authors:** Fabrizio Spada, Tanya M Barnes, Kerryn A Greive

**Affiliations:** ^1^ Ego Pharmaceuticals Braeside Victoria Australia

**Keywords:** corticosteroid, eczema, mometasone furoate, psoriasis, seborrhoeic dermatitis

## Abstract

Derivatives of hydrocortisone, such as mometasone furoate, a (2′) furoate‐17 ester with chlorine substitutions at positions 9 and 21, have been designed to improve efficacy and reduce the incidence of adverse effects. An extensive literature search of MEDLINE, Embase and other databases was conducted to review the safety and efficacy of various formulations of topical mometasone furoate. Mometasone furoate exhibits high potency with greater anti‐inflammatory activity and a longer duration of action than betamethasone. In clinical trials, mometasone furoate shows comparable or significantly better efficacy, depending on the comparator, in all indications studied in both adults and children. It is well tolerated with only transient, mild to moderate local adverse effects. It is characterised by low systemic availability due to its high lipophilicity, low percutaneous absorption and rapid hepatic biotransformation, and consequently has no significant effect on the hypothalamic‐pituitary‐adrenal axis. The molecular biotransformation of mometasone furoate in the skin results in a lower affinity with dermal cells than epidermal cells, which contributes to its low atrophogenicity. Sensitisation to mometasone furoate is low. Overall, mometasone furoate is a highly efficacious potent corticosteroid with a low risk of both local and systemic adverse effects.

## Introduction

Since the introduction of hydrocortisone in 1952, topical corticosteroids have become the cornerstone of treatment for many inflammatory skin conditions due to their ability to reduce inflammation.^1^ Initial success with hydrocortisone spurred the development of new topical corticosteroids by modifying both the ring structure and side chains of the hydrocortisone molecule, leading to compounds with variable anti‐inflammatory potency and side‐effect profiles.^2^, ^3^ These topical corticosteroids are classified in order of decreasing potency into four classes in Australia and the UK and seven classes in the USA, as per the Stoughton–Cornell classification (Table 1).^4^

**Table 1:** Classification of the potency of commonly used topical corticosteroid preparations available in Australia. Adapted from Carlos and colleagues^4^

	Formulations available			
	Ointment	Cream	Lotion	Other
Super potent (class 1 USA, class 1 UK)				
Betamethasone dipropionate 0.05% in optimised vehicle	**×**	**×**		
Clobetasol propionate 0.05%				Shampoo
High potency (class 2/3 USA, class II UK)				
Betamethasone dipropionate 0.05%	**×**			
Betamethasone valerate 0.1%	**×**	**×**		
Mometasone furoate 0.1%	**×**	**×**	**×**	Hydrogel
Moderate potency (class 4/5 USA, class III UK)				
Betamethasone dipropionate 0.05%		**×**	**×**	
Betamethasone valerate 0.02–0.05%	**×**	**×**		
Triamcinolone acetonide 0.02%	**×**	**×**		
Methylprednisolone aceponate 0.1%	**×**	**×**	**×**	
Clobetasone 0.05%		**×**		
Desonide 0.05%			**×**	
Low potency (class 6/7 USA, class IV UK)				
Hydrocortisone 0.5–1%		**×**		Spray
Hydrocortisone acetate 0.5–1%	**×**	**×**		

Topical corticosteroids have been associated with both local (more frequent) and systemic (infrequent) adverse effects including cutaneous atrophy, telangiectasia, striae, steroid rosacea and perioral dermatitis, hypothalamic‐pituitary‐adrenal axis suppression and skin infections.^4^, ^5^ The potential for side‐effects is often associated with the prolonged or widespread use of topical corticosteroids and usually correlates with increased clinical potency.^4^, ^5^ The risk is much less when topical corticosteroids are used appropriately as per guidelines.

Topical corticosteroids are available in a variety of vehicles such as ointments, creams, lotions and gels.^4^ Recent advances in formulation technology have resulted in the development of hydrogel vehicles that are water‐based, alcohol‐free, non‐irritating, non‐greasy and moisturising.^6^ The pharmaceutical formulation of topical corticosteroids has a great influence on whether or not it penetrates the stratum corneum, and consequently on the local bioavailability and efficacy of the steroid.^4^ Further, the cosmetic aspect of treatment has been found to have a major impact on patients’ adherence, and therefore on the efficacy of the corticosteroid.^6^, ^7^

Topical mometasone furoate (0.1% w/w) is classified as a high potency corticosteroid and has been available in Australia since 1987 for S4 (prescription‐only medicine in Australia) topical use as a cream, an ointment and a lotion (Table 1).

An extensive literature search of MEDLINE, Embase, the Cochrane Library and Dialog databases was conducted to March 2017. Only articles of high quality directly pertaining to the safety and efficacy of topical mometasone furoate of various formulations in relation to other corticosteroids in the treatment of psoriasis, eczema, atopic dermatitis and seborrhoeic dermatitis were included. Abstracts and studies relating to allergic contact dermatitis, vitiligo, phimosis, acute radiation dermatitis, lichen sclerosus, melasma, chronic idiopathic urticaria and alopecia areata were not included.

## Mometasone Furoate Chemical Structure

**Figure 1:**
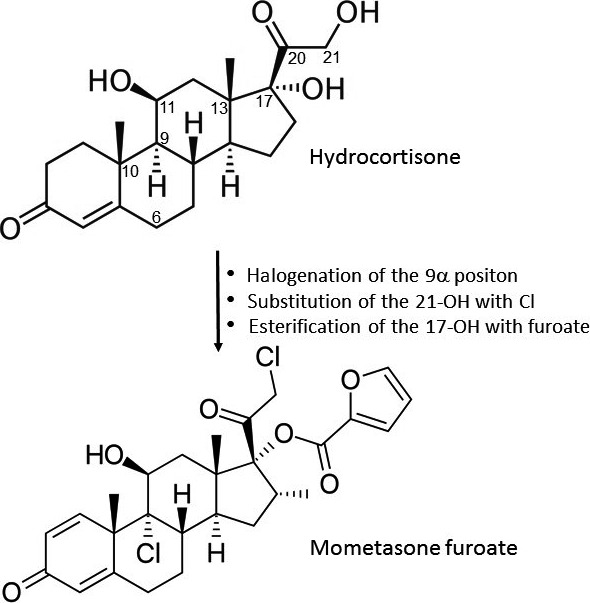
Changes in hydrocortisone leading to the formation of mometasone furoate. Cl, chlorine.

Mometasone furoate (9α,21‐dichloro‐11β,17α –dihydroxy‐16α‐methyl‐pregna‐1,4‐diene‐3,20‐dione‐17‐(2′) furoate) is the 17‐ester of the 16α‐methyl analogue of beclomethasone (Fig. 1) with an empirical formula of C_27_H_30_CI_2_O_6_ and a molecular weight of 521.4.^5^, ^7^ Halogenation of the 9α‐position, the substitution of the 21‐OH by chlorine and the esterification of the 17‐OH with furoate considerably increases the binding affinity of mometasone furoate to the corticosteroid receptor.^5^, ^7^ The ester hydrolysis biotransformation reduces the receptor binding during the passage through the skin, with mometasone furoate displaying a lower affinity to dermal than epidermal cells.^5^, ^7^ Further, chlorination at the 21 position and esterification at the 17 position increases the lipophilicity of mometasone furoate.^5^, ^7^ This allows mometasone furoate to permeate the stratum corneum and reach a therapeutic concentration in the skin, but without passing into the systemic circulation, thus avoiding further toxicity.^5^, ^7^

## Mechanism of Action

Mometasone furoate exhibits anti‐inflammatory, anti‐pruritic and vasoconstrictive properties.^5^, ^7^ Topical mometasone furoate 0.1% ointment has been shown to have a twofold to fourfold greater anti‐inflammatory activity and a longer duration of action than both topical betamethasone dipropionate 0.05% and betamethasone valerate 0.1% ointments, and its activity is similar to methylprednisolone aceponate 0.1% ointment in suppressing erythema induced by UVB light in healthy volunteers.^5^

The precise mechanism by which corticosteroids exert their anti‐inflammatory effect is unknown. In general, corticosteroids bind to specific corticosteroid receptors present in the cytoplasm.^4^, ^5^ The newly formed corticosteroid‐corticosteroid receptor complex translocates into the cell nucleus where it binds to corticosteroid response elements in the promoter region of the target genes, resulting in the regulation of gene expression.^5^ This results in the synthesis of certain anti‐inflammatory proteins, while inhibiting the synthesis of certain inflammatory mediators.^5^ Specifically, mometasone furoate is thought to act by inhibiting the arachidonic acid pathway, significantly reducing leukotriene production, inhibiting the production of inflammatory cytokines and growth factors, and decreasing the expression of adhesion molecules.^5^

## Pharmacokinetics

The extent of percutaneous absorption of topical corticosteroids depends on the vehicle, the condition of the epidermal barrier and the use of occlusive dressings.^7^ The high lipophilicity of mometasone furoate ensures that it binds very strongly with its receptor in the skin, thereby limiting its potential for systemic effects.^5^, ^7^ Only very minimal amounts of mometasone furoate have been shown to reach the systemic circulation following topical administration.^5^, ^7^ For example, following a single application of radiolabelled mometasone furoate 0.1% cream or ointment to the skin of healthy volunteers for 8 h, approximately 0.4% or 0.7%, respectively, of the applied dose was found to be absorbed systemically, with 94% of the total dose remaining unabsorbed on the skin and approximately 1.6% diffusing into the skin over the 5‐day study period.^5^, ^7^ When 10 g/day of mometasone furoate 0.1% ointment was applied under occlusion for 20 h/day for 5 days in healthy volunteers, plasma concentrations of mometasone furoate peaked at 130 ng/L and declined rapidly to 15 ng/L after 72 h. Only 0.00076% of the entire dose was excreted in the urine, and no metabolites were detected in the plasma.^5^, ^7^ Any mometasone furoate that does reach systemic circulation has a low resorption rate and undergoes rapid biotransformation in the liver.^5^, ^7^

## Clinical Safety

In clinical studies designed to investigate the effect of topical mometasone furoate on the hypothalamic‐pituitary‐adrenal axis in healthy adult volunteers (Table 2), no clinically significant decrease in serum cortisol levels were observed when 10 g/day mometasone furoate 0.1% ointment was applied with occlusion for up to 20 h/day for 5 days.^8^ A decrease in serum cortisol levels was found when 30 g/day mometasone furoate 0.1% ointment was applied to 60% of the body surface with occlusion for 22 h/day for 5 days. However, this decrease was found to be equivalent to that observed for methylprednisolone aceponate 0.1% ointment.^9^ In another study 16 g/day mometasone furoate 0.1% ointment applied with occlusion for 11 h/day for 5 days was found to produce a significant decrease in plasma cortisol levels, which was greater than that observed for hydrocortisone butyrate 0.1%.^10^ Although some decreases in serum cortisol levels were observed in these studies, no symptoms of hypothalamic‐pituitary‐adrenal axis suppression such as fatigue, anorexia, nausea, vomiting or weight loss, was observed in any of the volunteers.^5^, ^8^, ^9^, ^10^ In further clinical studies in patients with psoriasis,^11^ adult patients with atopic dermatitis^12^ children with atopic dermatitis,^13^, ^14^ and in adults with various other dermatoses,^2^ mometasone furoate 0.1% cream applied once daily for up to 12 weeks was not associated with any significant change in mean cortisol levels from baseline.

**Table 2:** Effect of topical mometasone furoate 0.1% ointment and cream on serum cortisol levels and its potential to cause skin atrophy

Reference	Trial design		Treatment	Duration	Patients treated (evaluated) (*n*)	Comparator potency^3^	Safety (*n* of patients)
Effect on serum cortisol levels							
Higashi and colleagues^8^	nb		MF 0.1% ung 10 g/day (20 h/day) oc	5 days	5 (5)	NA	No effect on serum cortisol levels
Bressinck and colleagues^11^, ^a^	r, db, pg		MF 0.1% ung 15 g od	3 weeks	24 (24)		Slight change in plasma cortisol level from baseline for both MF and HYD, which was NS from each other; MF ≡ HYD
			HYD 1.0% ung 15 g od		24 (24)	Low	
Kecskés and colleagues^9^	r, db		MF 0.1% ung 30 g od (22 h/day), 60% body oc	5 days	11 (11)		Both MF and MPA decreased serum cortisol levels to a similar extent; MF ≡ MPA
			MPA 0.1% ung 30 g od (22 h/day), 60% body oc		10 (10)	Moderate	
Visscher and colleagues^10^	r, o, co		MF 0.1% cr 16 g/day (11 h/day) oc	5 days	12 (12)	Moderate	Both MF and HYDB produced significant suppression of plasma cortisol concentrations during treatment, however complete recovery of the adrenal function took place once treatment ceased; MF > HYDB
			HYDB 0.1% cr 16 g/day (11 h/day) oc		12 (12)		
Effect on the skin							
Brasch in Prakash^5^		o	MF 0.1% cr od	52 weeks	6	NA	No clinical or histological signs of skin atrophy seen
Katz and colleagues^18^, ^a^		bpc	MF 0.1% ung od	6 weeks	51 (51)		MF: mild skin thinning (1), moderate telangiectasia (1)
			HYD 1.0% ung od		51 (51)	Low	HYD: mild skin thinning (1); MF ≡ HYD
Kerscher and colleagues^16^		r, db	MF 0.1% ung od	6 weeks	12		All treatments reduced skin thickness over 6 weeks, however this reduction was NS compared to baseline; PRD ≡ MF ≥ HYD ≡ V
			HYD 1.0% ung bid		12	Low	
			PRD 0.25% ung bid		12	Moderate	
			V		12	NA	
Hoffmann and colleagues^15^		r, db, ic	MF cr 200 mg od oc	3 weeks	10 (10)		NS changes in skin thickness as determined by ultrasound for all groups
			HYD 0.1% cr 200 mg od oc			Low	
			MPA cr 200 mg od oc			Moderate	No signs of skin atrophy for all groups; MF ≡ MPA ≡ HYD ≡ V
			V (MF, MPA concentration unknown)			NA	
Kecskés and colleagues^9^		r, db, ic	MF 0.1% ung 3 × /week oc	6 weeks	20 (20)		MF: pronounced skin atrophy (10), moderate skin atrophy (8), slight skin atrophy (2), very pronounced telangiectasia (5), pronounced telangiectasia (12), moderate telangiectasia (2), slight telangiectasia (1)
			MPA 0.1% ung 3×/week oc		20 (20)	Moderate	MPA: slight skin atrophy (15), no skin atrophy (5), moderate telangiectasia (3), slight telangiectasia (13), no telangiectasia (4)
			V		20 (20)	NA	V: slight skin atrophy (3), telangiectasia (0); MF > MPA
Korting and colleagues^1^		r, db	MF 0.1% ung bid	6 weeks	24 (22)		MF: skin thickness reduced by 17%; skin atrophy (2); telangiectasia (2)
			BMV 0.1% ung bid		24 (23)	High	BMV: skin thickness reduced by 24%; skin atrophy (2); telangiectasia (2)
			PRD 0.25% ung bid		24 (23)	Moderate	PRD: skin thickness reduced by 13%; skin atrophy (0); telangiectasia (0)
			V		24 (23)	NA	V: skin atrophy (0); telangiectasia (0); BMV > MF > PRD > V
Koivukangas and colleagues^17^		o, db	MF 0.1% cr od	1 weeks	15 (15)		No detectable effect on skin thickness was seen; MF ≡ BMV
			BMV 0.1% cr bid			High	
			V			NA	No difference between MF and BMV in their ability to reduce collagen synthesis

a Patients with psoriasis. bid, twice daily; BMV, betamethasone valerate; bpc, bilateral paired comparison; co, cross‐over; cr, cream; db, double blind; HYD, hydrocortisone; HYDB, hydrocortisone butyrate; ic, intra‐individual comparison; MF, mometasone furoate; MPA, methylprednisolone aceponate; NA, not applicable; nb, nonblind; NS, not significant; o, open label; oc, with occlusion; od, once daily; pg, parallel group; PRD, prednicarbate; r, randomised; ung, ointment; V, vehicle.

In clinical studies designed to investigate the atrophogenic potential of topical mometasone furoate 0.1% (Table 2), no clinical or histological signs of skin atrophy were observed in six volunteers after 12 months of a once‐daily application of mometasone furoate 0.1% cream.^5^ Further clinical trials of up to 6 weeks in healthy adult volunteers^15^, ^16^, ^17^ and patients with psoriasis^18^ have found the atrophogenic potential of mometasone furoate 0.1% ointment or cream to be low. However, in two studies, mometasone furoate 0.1% ointment used for 6 weeks with occlusion in healthy adult volunteers was associated with a significantly greater incidence and severity of skin atrophy and telangiectasia than methylprednisolone aceponate 0.1% ointment^9^ and prednicarbate 0.1% ointment,^1^ but it was not as pronounced as it was for betamethasone valerate 0.1% ointment.^1^

In clinical trials (presented in Supplementary Table S1, S2, and S3), a once‐daily application of various topical formulations of mometasone furoate 0.1% applied without occlusion was found to be generally well tolerated, regardless of the patient's age or dermatological condition. Adverse reactions reported in <5% of patients include transient and mild to moderate pruritus, burning, stinging, folliculitis, dryness, acneiform eruptions, and signs of mild skin atrophy and telangiectasia. Less common adverse reactions found in <1% of patients include erythema, oedema, fissures, urticaria, disease exacerbation, pimples, papular and pustular formations. These adverse events were no more pronounced than those observed for other corticosteroids, even those of low potency.^5^, ^18^ A few cases have been reported of severe side‐effects. However, these are very rare and have been associated with patient abuse or the long‐term use of topical mometasone furoate.^19^

Patch test studies have shown that topical mometasone furoate is associated with a negligible risk of primary contact sensitisation and allergic cross‐reaction, even in patients known to be hypersensitive to corticosteroids.^5^

## Safety in Pregnancy and Breastfeeding

Topical mometasone furoate 0.1% has been classified as a category B3 drug in Australia. There are no adequate and well‐controlled studies of the teratogenic effects of mometasone furoate in pregnant women.^4^ It should therefore be used with caution during pregnancy and only if the potential benefit to the patient outweighs the potential risk to the foetus.^4^ Further, high‐potency corticosteroids should not be used on pregnant patients in large amounts or for prolonged periods of time.

Systemically administered corticosteroids are secreted into breast milk but the quantities are too low to have a deleterious effect on the infant. It is not known whether topically applied mometasone furoate is absorbed in sufficient quantities to produce detectable levels in breast milk.^4^ Therefore, mometasone furoate should be used with caution during breastfeeding.^4^ Temporary cessation of breastfeeding during treatment should also be considered.

## Clinical Efficacy

Supplementary Table S1 shows the efficacy of mometasone furoate 0.1% ointment compared with other corticosteroid ointments observed in clinical trials, as determined by the percentage improvement from baseline in total disease sign or symptom scores. The efficacy of mometasone furoate 0.1% ointment in patients with moderate to severe psoriasis vulgaris (*n* = 48–243) in comparative 2–8 week trials was significantly greater than that of the vehicle,^3^ mildly potent hydrocortisone 1.0% ointment applied once daily,^11^, ^18^ moderately potent fluocinolone acetonide 0.025% ointment applied thrice daily^20^ and several other highly potent topical corticosteroids ointments applied twice daily, including triamcinolone acetonide 0.1%,^20^ fluticasone propionate 0.005%^21^ and betamethasone valerate 0.1%^3^, ^22^, ^23^ in patients aged 12 years or older. However, there was no significant difference in patients with psoriasis^24^ or atopic dermatitis^25^ treated with either mometasone furoate 0.1% ointment or highly potent betamethasone dipropionate 0.05% ointment for up to 4 weeks. In addition, there was no significant difference in treatment outcomes when topical mometasone furoate 0.1% ointment was applied either once or twice daily in patients with psoriasis for up to 15 days.^26^

Supplementary Table S2 shows clinical trials comparing the efficacy of once‐daily mometasone furoate 0.1% cream to other corticosteroid creams as determined by the percentage improvement from baseline in total disease sign and symptom scores. In adults, the efficacy of mometasone furoate 0.1% cream applied once daily in patients with moderate to severe psoriasis vulgaris (*n* = 132–218) in comparative 3 week trials was significantly greater than that of moderately potent fluocinolone acetonide 0.025% cream applied thrice daily^20^ and similar to that of high‐potency triamcinolone acetonide 0.1% ointment applied twice daily.^20^ Similarly, in adults with atopic dermatitis or seborrhoeic dermatitis, 0.1% mometasone furoate cream was significantly superior to less potent corticosteroids, including hydrocortisone butyrate^12^ and hydrocortisone 1.0%^27^ cream. Several clinical trials have examined the efficacy of mometasone furoate 0.1% cream in adult patients (*n* = 34–216) with various corticosteroid‐responsive dermatoses from 2–12 weeks. Mometasone furoate 0.1% cream applied once daily was found to have an efficacy similar to corticosteroids with similar potency applied twice daily, such as betamethasone dipropionate 0.05%^2^ and betamethasone valerate 0.1%^28^, ^29^ creams. Mometasone furoate 0.1% cream was also found to be significantly more efficacious than corticosteroids of moderate potency applied twice daily for up to 3 weeks, such as hydrocortisone butyrate 0.1% cream,^30^, ^31^ but it was significantly inferior to super‐potent clobetasol propionate 0.05% cream in patients with eczema.^32^

In children aged between 6 months and 12 years with atopic dermatitis, mometasone furoate 0.1% cream applied once daily was found to be significantly superior to less potent corticosteroids applied twice daily, such as hydrocortisone 1.0%,^13^ clobetasone 0.05%^14^ and hydrocortisone valerate 0.2%^33^ creams. However, mometasone furoate 0.1% cream was found to have similar efficacy to clobetasone butyrate 0.05% in children with mixed dermatoses.^34^ In further studies it was shown that a regimen of mometasone furoate 0.1% cream applied 2 or 3 days/week for up to 36 weeks, and 2 days/week for 26 weeks was prophylactic in both adults with eczema^35^ and children with atopic dermatitis.^36^ Recently, new cream formulations of mometasone furoate have become available, which have been shown to be bioequivalent to the older preparations.^7^, ^37^, ^38^, ^39^

The efficacy of mometasone furoate 0.1% lotion applied once daily to patients with moderate to severe scalp psoriasis aged ≥12 years (*n* = 192–203) in comparative 3‐week trials was significantly greater than that of other highly potent topical corticosteroids lotions including triamcinolone acetonide 0.1%^40^ and betamethasone valerate 0.1%^41^ applied twice daily (Supplementary Table S3). A formulation of mometasone furoate 0.1% using a novel water‐based, alcohol‐free, non‐irritating, non‐greasy and moisturising hydrogel as a vehicle has been developed for use in Australia. Clinical trials have not been conducted with mometasone furoate 0.1% hydrogel; however, mometasone furoate 0.1% hydrogel formulation has been shown to be bioequivalent to mometasone furoate 0.1% lotion.^6^

## Conclusion

The effect of topical mometasone furoate 0.1% in various topical preparations has been well studied over many years. In clinical trials the efficacy of mometasone furoate 0.1% ointment, cream and lotion applied once daily to patients with a variety of inflammatory skin conditions including psoriasis, eczema, atopic dermatitis and seborrhoeic dermatitis for between 2–12 weeks, was found to be significantly superior to twice‐daily applications of less potent corticosteroids of similar formulations, and it was comparable to or significantly superior to that of several other highly potent corticosteroids of a similar formulation that required application twice or thrice daily, regardless of the patients’ age.

Although mometasone furoate 0.1% demonstrates greater anti‐inflammatory activity and a longer duration of action than betamethasone relative to other topical corticosteroids with a similar or weaker potency, topical formulations of mometasone furoate 0.1% have been shown to be associated with a low risk of corticosteroid‐related adverse events, such as skin atrophy and other local events, and to have a very limited potential to induce systemic adverse effects, including hypothalamic‐pituitary‐adrenal axis suppression.

## Supporting information

Table S1 Clinical trials examining the comparative safety and efficacy of mometasone furoate 0.1% ointment versus other corticosteroids in the management of patients with psoriasis vulgaris and atopic dermatitis.

Table S2 Clinical trials examining the comparative safety and efficacy of mometasone furoate 0.1% cream versus other corticosteroids in the management of patients with psoriasis vulgaris, atopic dermatitis, seborrhoeic dermatitis, eczema and other corticosteroid‐responsive dermatoses.

Table S3 Clinical trials examining the comparative safety and efficacy of mometasone furoate 0.1% lotion versus other corticosteroids in the management of patients with scalp psoriasis.
