# Supplementary material for: Comparative safety and efficacy of topical mometasone furoate with other topical corticosteroids
Source: Australas J Dermatol. 2018 Feb 7;59(3):e168–74. doi: 10.1111/ajd.12762 (PMC6099284; doi:10.1111/ajd.12762)
Supplement: Supplementary file 1 — Table S1 Clinical trials examining the comparative safety and efficacy of mometasone furoate 0.1% ointment versus other corticosteroids in the management of patients with psoriasis vulgaris and atopic dermatitis. [file AJD-59-e168-s001.docx]

**Table S1** Clinical trials examining the **c**omparative safety and efficacy of mometasone furoate 0.1% ointment versus other corticosteroids in the management of patients with psoriasis vulgaris and atopic dermatitis

| **Reference** | **Trial design** | **Treatment** | **Duration (weeks)** | **Number of patients treated (evaluated)** | **Comparator potency^3^** | **Efficacy: Mean % improvement in TSSS at endpoint**  **(unless specified)** | **Safety (number of patients shown in parentheses)** |
| --- | --- | --- | --- | --- | --- | --- | --- |
| **Moderate to severe psoriasis vulgaris** | | | | | | | |
| Medansky *et al*^3^ | r, db, pg, mc | MF 0.1% ung bid  V | 3 | 58 (50)  57 (45) | NA | 58**  36 | MF AE: mild burning or stinging for 10-15 min (2), pruritus for <10 min (3), severe burning for 5 min (1), skin atrophy (0).  V AE: were qualitatively the same as for MF, and were of mild severity in most instances. |
| Bressinck *et al*^11^ | r, db, pg | MF 0.1% ung 15 g od  HYD 1.0% ung 15 g od | 3 | 24 (24)  24 (24) | Low | 47**  ≤12 | Slight change in plasma cortisol level from baseline for both MF and HYD, which were NS from each other; MF≡HYD.  MF AE: moderate burning (1), mild telangiectasia (2).  HYD AE: mild itching (1). |
| Katz *et al*^18^ | bpc | MF 0.1% ung od  HYD 1.0% ung od | 6 | 51 (51)  51 (51) | Low | 60***  38 | MF: mild skin thinning (1), moderate telangiectasia (1).  HYD: mild skin thinning (1); MF≡HYD.  No other AE. |
| Medansky *et al*^20^ | r, sb, pg, mc | MF 0.1% ung od  FLU 0.025% ung tid | 3 | 112 (112)  107 (107) | Moderate | 58**  41 | MF AE: burning (<1%), itching (<2%), dryness (<0.2%), pimples (<0.2%), erythema (<0.2%), skin thinning (0.9%).  FLU AE: burning (<1%), itching (<2%), dry hair (<0.2%), pimples (<0.2%), erythema (<2%), fissures (<0.2%), psoriasis flare (<0.2%), urticaria (<0.2%), increased erythema (<0.2%), increased edema (<0.2%). |
| Medansky *et al*^20^ | r, sb, pg, mc | MF 0.1% ung od  TRI 0.1% ung bid | 3 | 98 (98)  97 (97) | High | 60**  37 | MF AE: burning (<1%), itching (<2%), dryness (<0.2%), pimples (<0.2%), erythema (<0.2%), skin thinning (<0.8%).  TRI AE: burning (<3%), itching (<6%), dryness (<0.6%), stinging (<0.6%), skin thinning (<2.5%). |
| De Villez *et al*^21^ | r, eb, pg, mc | MF 0.1% ung od  FLUP 0.005% ung bid | ≤3 | 128 (122)  129 (121) | High | 67**  56 | 19 AE for MF: mild to moderate stinging, itching and burning (5), mild skin atrophy (2), mild to moderate headache (5), unaccounted (7).  15 AE for FLUP: mild to moderate stinging, itching and burning (4), mild skin atrophy (3), mild to moderate headache (3), unaccounted (5). |
| Medansky *et al*^3^ | r, db, bpc | MF 0.1% ung bid  BMV 0.1% ung bid | 2 | 30 (30)  30 (30) | High | 52**  40 | No local or systemic AE reported for either MF or BMV. |
| Svensson *et al*^22^ | r, sb, pg, mc | MF 0.1% ung od  BMV 0.1% ung bid | 8 | 35 (32)  37 (36) | High | 67*  51 | MF AE: mild skin thinning (1), mild stinging (1).  BMV AE: mild skin thinning (1), mild loss of skin elasticity (1). |
| Rosenthal *et al*^23^ | r, tpb, pg, mc | MF 0.1% ung od  BMV 0.1% ung bid | 3 | 54 (52)  54 (52) | High | 74*  57 | MF AE: mild burning (2), severe pruritus (1), mild signs of atrophy (6).  BMV AE: mild burning (1), moderate pruritus (2), severe pruritus (1), mild sensitivity of fingertip to BMV (1), mild signs of skin atrophy (3). |
| Peharda *et al*^24^ | r, sb, pg | MF 0.1% ung od  BMD 0.05% ung bid | 4 | 30 (28)  30 (27) | High | 86% NS  85%  cured or had good to moderate improvement | MF AE: mild skin atrophy (1), irritation (1).  BMD AE: skin atrophy (4), irritation (2). |
| Singh *et al*^26^ | r, db, bpc | MF 0.1% ung od  MF 0.1% ung bid | ≤15 days | 68 (64)  68 (64) | NA | 66% NS  77%  complete clearance | ND |
| **Atopic dermatitis** | | | | | | | |
| Marchesi *et al*^25^ | r, tpb, pg | MF 0.1% ung od  BMD 0.05% ung bid | ≤3 | 30 (30)  30 (30) | High | 100% NS  100%  successfully treated | MF AE: mild telangiectasia (4).  BMD AE: mild telangiectasia (5), skin alterations accompanied by a loss of skin marks and reduced skin elasticity (1). |

**P* < 0.05, ***P* < 0.01, ****P* < 0.001 versus comparator. AE, adverse events; bid, twice daily; BMD, betamethasone dipropionate; BMV, betamethasone valerate; bpc, bilateral paired comparison; db, double blind; eb, evaluator blind; FLU, fluocinolone acetonide; FLUP, fluticasone propionate; HYD, hydrocortisone; mc, multicentre; MF, mometasone furoate; NA, not applicable; ND, not determined; NS, not significant; o, open; od, once daily; p, prospective; pg, parallel group; r, randomised; sb, single blind; tbp, third party blind; tid, three times daily; TRI, triamcinolone acetonide; TSSS, total sign and symptom severity score; ung, ointment; V, vehicle.
