# Supplementary material for: Comparative safety and efficacy of topical mometasone furoate with other topical corticosteroids
Source: Australas J Dermatol. 2018 Feb 7;59(3):e168–74. doi: 10.1111/ajd.12762 (PMC6099284; doi:10.1111/ajd.12762)
Supplement: Supplementary file 2 — Table S2 Clinical trials examining the comparative safety and efficacy of mometasone furoate 0.1% cream versus other corticosteroids in the management of patients with psoriasis vulgaris, atopic dermatitis, seborrhoeic dermatitis, eczema and other corticosteroid‐responsive dermatoses. [file AJD-59-e168-s002.docx]

**Table S2** Clinical trials examining the comparative safety and efficacy of mometasone furoate 0.1% cream versus other corticosteroids in the management of patients with psoriasis vulgaris, atopic dermatitis, seborrheic dermatitis, eczema and other corticosteroid responsive dermatoses

| **Reference** | **Trial design** | **Treatment** | **Duration (weeks)** | **Number of patients treated (evaluated)** | **Comparator potency^3^** | **Efficacy: Mean % improvement in TSSS at endpoint**  **(unless specified)** | **Safety (number of patients shown in parentheses)** |
| --- | --- | --- | --- | --- | --- | --- | --- |
| **Moderate to severe psoriasis vulgaris** | | | | | | | |
| Medansky *et al*^20^ | r, sb, pg, mc | MF 0.1% cr od  FLU 0.025% cr tid | 3 | 109 (109)  109 (109) | Moderate | 51***  29 | MF AE: burning (<1%), itching (<2%), dryness (<0.2%), pimples (<0.2%), erythema (<0.2%), skin thinning (0.9%).  FLU AE: burning (<1%), itching (<2%), dry hair (<0.2%), pimples (<0.2%), erythema (<2%), fissures (<0.2%), psoriasis flare (<0.2%), urticaria (<0.2%), increased erythema (<0.2%), increased edema (<0.2%). |
| Medansky *et al*^20^ | r, sb, pg, mc | MF 0.1% cr od  TRI 0.1% cr bid | 3 | 66 (66)  66 (66) | High | 54 NS  51 | MF AE: burning (<1%), itching (<2%), dryness (<0.2%), pimples (<0.2%), erythema (<0.2%), skin thinning (<0.8%).  TRI AE: burning (<3%), itching (<6%), dryness (<0.6%), stinging (<0.6%), skin thinning (<2.5%). |
| **Atopic dermatitis** | | | | | | | |
| Hoybye *et al*^12^ | r, sb, pg, mc | MF cr od for 3 wk and od first 3 days/wk for 3 wk  HYDB cr bid for 3 wk and bid for first 3 days/wk for 3 wk  (concentrations unknown) | 6 | 49 (48)  45 (38) | Moderate | 85%**  71%  successfully treated | No skin atrophy seen for MF or HYDB.  No change in plasma cortisol levels; MF≡HYDB.  MF AE: all mild AE including stinging, burning, itching, dryness, acne, folliculitis, hair growth.  HYDB AE: all mild AE including stinging, burning, itching, dryness, acne, folliculitis, hair growth. |
| **Seborrheic dermatitis** | | | | | | | |
| Medansky *et al*^27^ | r, tpb, pg, mc | MF 0.1% cr od  HYD 1.0% cr bid | 6 | 59 (59)  58 (58) | Low | 97*  91 | MF AE: mild papules (1), moderate papules (1), moderate eyelid infection (1), mild pustules (1), mild acneform rash (1), mild stinging (1), 1-2 of the signs of skin atrophy (6).  H AE: mild papules (1), 1-2 of the signs of skin atrophy (2) |
| **Eczema** | | | | | | | |
| Veien *et al*^35^ | p, r, o | MF 0.1% cr od  for 3 to 9 weeks then randomized to:  MF 0.1% cr 3×/wk  MF 0.1% cr 2×/wk  Emollients only | 30 | 120 (106)  35  37  34 | NA | 83%  68%  26%  no reoccurrence during maintenance phase | Mild skin atrophy was noted at some point in the study for 10 patients; 3 had atrophy at the onset which disappeared during the study (1 in 3×/wk group, 2 in 2×/wk group) and 5 had mild skin atrophy at the final visit (2 in 3×/wk group, 3 in 2×/wk group). |
| Goh *et al*^32^ | o, r, tpb, lrs | MF 0.1% cr od  CLOBP 0.05% cr bid | 3 | 60 (58) | Super | 28%  71%**  cured or marked clearance (>75%) | No AE reported for both MF and CLOBP. |
| **Mixed corticosteroid responsive dermatoses including allergic contact dermatitis, atopic dermatitis, psoriasis and seborrheic dermatitis** | | | | | | | |
| Gip *et al*^30^ | r, tpb, pg, mc | MF 0.1% cr od  HYDB 0.1% cr bid | ≤3 | 107 (107)  109 (109) | Moderate | 86*  77 | No skin atrophy observed for either MF or HYDB.  MF AE: mild burning (1), moderate burning (2).  HYDB AE: moderate folliculitis (2). |
| Fowler *et al*^31^ | r, db, lrs, mc | MF 0.1% cr bid  HYDB 0.1% cr bid | 2 | 34 (31) | Moderate | 37 NS  42 | NO AE reported for MF or HYDB. |
| Kelly *et al*^2^ | r, sb, pg, mc | MF 0.1% cr od  BMD 0.05% cr bid | ≤12 | 33 (22)  34 (30) | High | 74.4 NS  80.1 | Plasma cortisol levels remained within normal parameters at all times: MF≡BMD.  MF AE: mild to moderate AE (6), slight skin atrophy (2), moderate skin atrophy (2).  BMD AE: moderate AE (1), severe AE (2), barely detectable skin atrophy (1), mild skin atrophy (6), mild to moderate skin atrophy (3), moderate skin atrophy (1). |
| Viglioglia *et al*^28^ | r, ib, pg | MF 0.1% cr od  BMV 0.1% cr bid | ≤3 | 39 (35)  38 (34) | High | 93.6 NS  96.5 | No skin atrophy or AE observed for either MF or BMV. |
| Wishart *et al*^29^ | r, pg, mc | MF 0.1% cr od  BMV 0.1% cr bid | 4 | 29 (28)  30 (30) | High | 93 NS  90 | No skin atrophy observed for either MF or BMV.  MF AE: mild AE (4).  BMV AE: mild AE (2). |
| **Atopic dermatitis in children (aged between 6 months and 12 years)** | | | | | | | |
| Vernon *et al*^13^ | r, db, pg, mc | MF 0.1% cr od  HYD 1.0% cr bid | ≤6 | 24 (23)  24 (19) | Low | 95**  75 | No skin atrophy observed for either MF or HYD.  No significant change in plasma cortisol levels from baseline for either MF or HYD; MF≡HYD.  MF AE: stinging (3).  HYD AE: molluscum contagiosum (1). |
| Rafanelli *et al*^14^ | r, tpb, pg | MF 0.1% cr od  CLOB 0.05% cr bid | 3 | 30 (30)  30 (30) | Moderate | 86.1**  66.1 | No skin atrophy or AE observed for either MF or CLOB.  Plasma cortisol levels remained within normal parameters at all times: MF≡CLOB. |
| Lebwohl *et al*^33^ | r, eb, pg, mc | MF 0.1% cr od  HYDV 0.2% cr bid | ≤3 | 111 (102)  112 (107) | Moderate | 87.2**  78.6 | No skin atrophy observed for either MF or HYDV.  MF AE: mild AE in 3.7% of patients.  HYDV AE: mild AE in 1.8% of patients. |
| Faergemann *et al*^36^ | o, mc | Phase 1:  MF 0.1% cr od  If score ≤3 then Phase 2:  MF 0.1% cr 2×/wk | ≤3  26 | 90  68 (68) | NA | Phase 2: 90% patients did not relapse under the prophylactic regimen. | Phase 2 MF AE: folliculitis (2), sensation of warmth (1), signs of skin atrophy (1). |
| **Mixed corticosteroid responsive dermatoses in children (aged between 6 months and 12 years)** | | | | | | | |
| Dominguez *et al*^34^ | r, sb, pg | MF 0.1% cr od  CLOB 0.05% cr bid | ≤3 | 31 (29)  32 (32) | Moderate | 91.9 NS  86.9 | No skin atrophy observed for either MF or CLOB.  MF AE: No AE reported.  CLOB AE: mild folliculitis (2), mild to moderate folliculitis (1) |

**P* < 0.05, ***P* < 0.01, ****P* < 0.001 versus comparator. AE, adverse events; bid, twice daily; BMD, betamethasone dipropionate; BMV, betamethasone valerate; CLOB, clobetasone butyrate; CLOBP, clobetasol propionate; cr, cream; db, double blind; eb, evaluator blind; FLU, fluocinolone acetonide; HYD, hydrocortisone; HYDB, hydrocortisone butyrate; HYDV, hydrocortisone valerate; ib, investigator blind; lrs, left-right sided; mc, multicentre; MF, mometasone furoate; NA, not applicable; NS, not significant; o, open; od, once daily; p, prospective; pg, parallel group; r, randomised; sb, single blind; tbp, third party blind; tid, three times daily; TRI, triamcinolone acetonide; TSSS, total sign and symptom severity score; wk, week.
