# Supplementary material for: Comparative safety and efficacy of topical mometasone furoate with other topical corticosteroids
Source: Australas J Dermatol. 2018 Feb 7;59(3):e168–74. doi: 10.1111/ajd.12762 (PMC6099284; doi:10.1111/ajd.12762)
Supplement: Supplementary file 3 — Table S3 Clinical trials examining the comparative safety and efficacy of mometasone furoate 0.1% lotion versus other corticosteroids in the management of patients with scalp psoriasis. [file AJD-59-e168-s003.docx]

**Table S3**  Clinical trials examining the comparative safety and efficacy of mometasone furoate 0.1% lotion versus other corticosteroids in the management of patients scalp psoriasis

| **Reference** | **Trial design** | **Treatment** | **Duration (weeks)** | **Number of patients treated (evaluated)** | **Comparator potency^3^** | **Efficacy: Mean % improvement in TSSS at endpoint** | **Safety (number of patients shown in parentheses)** |
| --- | --- | --- | --- | --- | --- | --- | --- |
| **Moderate to severe scalp psoriasis** | | | | | | | |
| Swinehart *et al*^40^ | tpb, pg, mc | MF 0.1% lot od  TRI 0.1% lot bid | 3 | 103 (99)  99 (93) | High | 78*  73 | 7 AE for MF including a stinging sensation on the scalp, facial tenderness and acne.  6 AE for TRI including folliculitis and pruritus. |
| Vanderploeg *et al*^41^ | r, tpb, pg, mc | MF 0.1% lot od  BMV 0.1% lot bid | 3 | (101)  (102) | High | 85***  70 | MF AE: itching (3), folliculitis (2), acne: 1, burning (1), tender scalp (1), skin atrophy (0)  BMV AE: itching (2), folliculitis (1), acne (1), burning/warmth (2), hair discoloration (1), stinging (3), skin atrophy (0) |

**P* < 0.05, ***P* < 0.01, ****P* < 0.001 versus comparator. AE, adverse events; bid, twice daily; BMV, betamethasone valerate; lot, lotion; mc, multicentre; MF, mometasone furoate; od, once daily; pg, parallel group; r, randomised; tbp, third party blind; TRI, triamcinolone acetonide; TSSS, total sign and symptom severity score.
